# Synthesis and Bioactivity Evaluation of Novel 2-Salicyloylbenzofurans as Antibacterial Agents

**DOI:** 10.3390/molecules22050687

**Published:** 2017-04-25

**Authors:** Phuong-Thuy T. Phan, Thu-Trang T. Nguyen, Hong-Nhung T. Nguyen, Bao-Khanh N. Le, Thao T. Vu, Dong C. Tran, Tuan-Anh N. Pham

**Affiliations:** 1 Department of Medicinal Chemistry, Faculty of Pharmacy, Ton Duc Thang University, Ho Chi Minh City 700000, Vietnam; 2 Department of Pharmaceutics, Faculty of Pharmacy, Da Nang University of Medical Technology and Pharmacy, Da Nang 550000, Vietnam; nguyenthithutrang184@gmail.com; 3 Department of Organic Chemistry, Faculty of Pharmacy, University of Medicine and Pharmacy at Ho Chi Minh City, Ho Chi Minh City 700000, Vietnam; hongnhungrose2193@gmail.com (H.-N.T.N.); lnbkhanh@gmail.com (B.-K.N.L.); 4 Department of Microbiology and Parasitology, Faculty of Pharmacy, University of Medicine and Pharmacy at Ho Chi Minh City, Ho Chi Minh City 700000, Vietnam; vuthao910@gmail.com (T.T.V.); trancatdong@gmail.com (D.C.T.)

**Keywords:** 2-salicyloylbenzofuran, benzofuran, antibacterial activity, MRSA, Rap–Stoermer condensation, Williamson synthesis

## Abstract

In order to discover new antibacterial agents, series of 2-salicyloylbenzofuran derivatives were designed, synthesized and evaluated for their antibacterial activities against three Gram-(+) strains (methicillin-sensitive *Staphylococcus aureus* (MSSA) ATCC 29213, methicillin-resistant *Staphylococcus aureus* (MRSA) ATCC 43300, and *Streptococcus faecalis* (*S. faecalis*) ATCC 29212) and one Gram-(−) strain (*Escherichia coli (E. coli)* ATCC 25922). The 2-salicyloylbenzofuran heterocycles were generated by Rap–Stoermer condensation of salicylaldehydes with phenacyl bromides and then converted to diverse *O*-ether derivatives by Williamson synthesis. The targeted products were screened for in vitro qualitative (zone of inhibition) and quantitative (MIC) antibacterial activities by agar well diffusion assay and agar dilution method. Amongst the compounds, those bearing carboxylic acid functional group were found to exhibit reasonable activity against Gram-(+) bacterial strains including *S. faecalis*, MSSA and MRSA with the most potent antibacterial agent **8h** (MICs = 0.06–0.12 mM). Besides, the 2-salicyloylbenzofurans partly displayed inhibitory activity against MRSA with the best MICs = 0.14 mM (**8f**) and 0.12 mM (**8h**). Finally, the antibacterial results preliminarily suggested that the substituent bearing carboxylic acid group at salicyloyl-C2 and the bromine atoms on the benzofuran moiety seem to be the functionality necessary for antibacterial activities.

## 1. Introduction

Drug resistant infections are complex problems that have been threatening the health care with related morbidity and mortality being on the rise worldwide. Moreover, infectious diseases have been extremely difficult to treat due to antibiotic resistant problem resulting in high extra in-hospital costs [1,2,3,4]. Therefore, the prevention and control of the resistance threats have been identified as global public health priorities that require actions across all government sectors and society [1,2,3,4]. To combat the increasingly serious threat, one of the most important main actions is to accelerate the discovery and development of new classes of antibacterial agents as well as exploring possible new drugs from existing classes of antibiotics that can effectively against the multidrug-resistant bacterial strains [1,2,4].

In the literature, we found that the naturally diaryl(heteroaryl)ketone scaffold such as pestalone [5], marinopyrrole A–F [6,7], pyrrolomycin C [8], pyrrolomycin J [9], and synthetic compounds related to pyrrolomycins including 3,4,5,3′,5′-pentabromo-2-(2′-hydroxybenzoyl)pyrrole [10] and fluorinated pyrrolomycines [11], displayed strong inhibitory activities against bacterial strains. Remarkably, these halogenated ketones showed potent antibacterial activity against MRSA [5,6,7,9,11], and vancomycin-resistant *Enterococcus faecium* [5] at low minimum inhibitory concentrations (MICs). The important characteristic features of these ketones for antibacterial activities are their high halogen (F, Cl, and Br) content and the presence of -OCH_3_/-OH substituent at the aroyl-C2 moiety [7,8,9,10,11].

**Figure 1:**
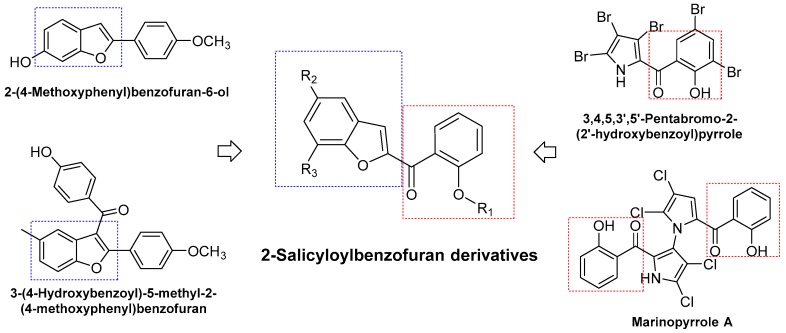
Design of novel 2-salicyloylbenzofuran derivatives as antibacterial agents.

Similarly, benzofuran is considered as one of the important heterocycles because of its diverse biological properties such as antibacterial, antitubercular, antiviral, anticancer, anti-inflammatory, etc. [12,13,14]. Many of the clinically approved drugs are mono-benzofuran derivatives as well as fused benzofurans with other heterocycles [13]. In particular, the antibacterial activities of benzofuran scaffold have been found very promising potential against Gram-(+) and Gram-(–) bacteria, including MRSA [12,13,14,15,16]. Several remarkable agents such as 3-(4-hydroxybenzoyl)-5-methyl-2-(4-methoxyphenyl)benzofuran, 2-(4-methoxyphenyl)benzofuran-6-ol have been reported in recent articles [15,16]. Therefore, in order to discover new antibacterial agents, series of 2-salicyloylbenzofuran derivatives were designed, synthesized and evaluated for their antibacterial activities against three Gram-(+) strains (MSSA ATCC 29213, MRSA ATCC 43300, *S. faecalis* ATCC 29212) and one Gram-(–) strain (*E. coli* ATCC 25922) (Figure 1).

## 2. Results and Discussion

### 2.1. Chemistry

**Scheme 1:**
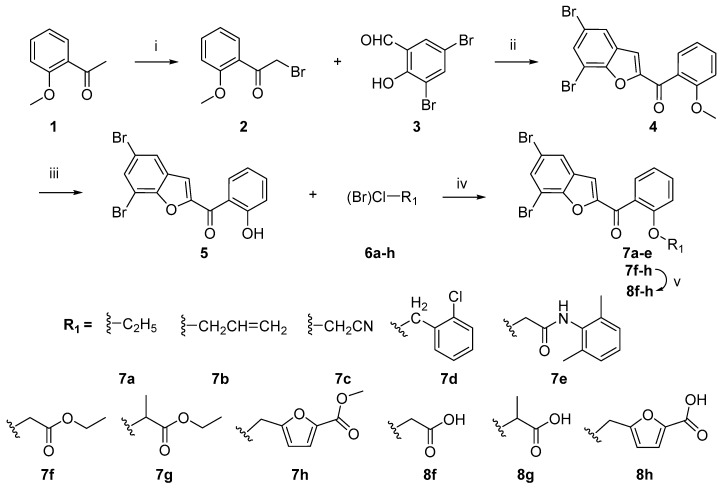
Synthesis of 5,7-dibromo-2-salicyloylbenzofuran *O*-ether derivatives **7a**–**h** and **8f**–**h**. Reagents and conditions: (i) NBS, PTSA, 60 °C, 15 min., 83%; (ii) DMAP, Na_2_CO_3_, H_2_O, 80 °C, 5 h, 72%; (iii) AlCl_3_, DCM, r.t., 3 h, 89%; (iv) K_2_CO_3_, acetone, reflux, 8 h, 60–87%; and (v) 1. NaOH, CH_2_Cl_2_/CH_3_OH (9:1), r.t., 1 h 2. HCl, 70–82%.

The *O*-ether derivatives of 5,7-dibromo-2-salicyloyl benzofuran **7a**–**h** and **8f**–**h** were prepared according to the synthetic Scheme 1. Bromination of starting material 2-methoxyacetophenone **1** using NBS as bromine source in the presence of *p*-toluenesulfonic acid (PTSA) gave 2-methoxyphenacyl bromide **2** [17] that was reacted with 3,5-dibromosalicyladehyde via Rap–Stoermer condensation reaction, using 4-dimethylaminopyridine (DMAP) as catalyst, to obtain 5,7-dibromo-2-(2-methoxybenzoyl)benzofuran **4** [18]. Demethylation of compound **4** by anhydrous AlCl_3_ in dried dichloromethane afforded 5,7-dibromo-2-salicyloylbenzofuran **5** as key intermediate in good yield [19,20]. *O*-ether derivatives of compound **5** were synthesized by Williamson reaction at 2-OH group of the salicyloyl moiety with diverse halogenated materials **6a**–**h** (for details see Section 3.2.4) using K_2_CO_3_ as a base in acetone gave the desired compounds **7a**–**h** [21]. Alkaline hydrolysis of esters **7f**–**h** in non-aqueous condition furnished the products **8f**–**h** as their respective carboxylic acid derivatives [22].

**Scheme 2:**
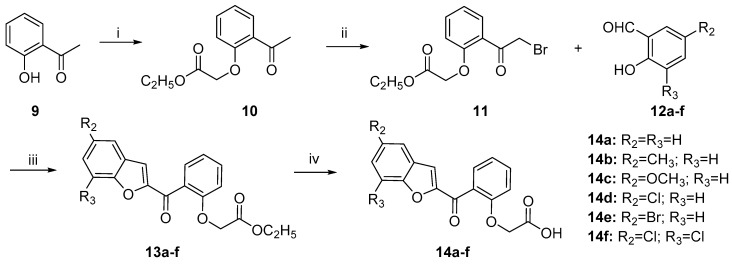
Synthesis of 2-(2-(benzofuran-2-carbonyl)phenoxy)ethanoic acid derivatives **14a**–**f**. Reagents and conditions: (i) **6f**, K_2_CO_3_, acetone, reflux, 8 h, 77%; (ii) NBS, PTSA, 60 °C, 15 min., 74%; (iii) DMAP, Na_2_CO_3_, H_2_O, 80 °C, 5 h, 57–72%; and (iv) 1. NaOH, CH_2_Cl_2_/CH_3_OH (9:1), r.t., 1 h 2. HCl, 66–81%.

To investigate the role of the substituents on the benzofuran moiety on the antibacterial activities, series of 2-(2-(benzofuran-2-carbonyl)phenoxy)ethanoic acid derivatives **14a**–**f** were synthesized according to the synthetic Scheme 2. The key intermediate **11** was prepared by *O*-etherification at 2-OH group of 2-hydroxyacetophenone **9** with ethyl chloroacetate **6f** [21] and then brominated by NBS in the presence of PTSA as catalyst [17]. The Rap–Stoermer condensation reaction was applied to generate the benzofuran heterocycles **13a**–**f** [18] that was subjected to alkaline hydrolysis in non-aqueous condition to obtain the final products **14a**–**f** [22].

In this study, the Rap–Stoermer condensation reaction was applied for the efficient generation of benzofuran heterocycle in 57–87% yields. The Williamson reaction was used to synthesize desired 2-salicyloylbenzofuran derivatives bearing diverse *O*-ether moieties in rather good yields (60–87%). It was found that the most preferable alkylating agents for ether formation are primary α-halogenated materials since the Williamson reaction using ethyl 2-bromopropanoate **6g**, a secondary α-halogenated material, gave ether product **7g** with the lowest yield (60%). The structures of synthesized 2-salicyloylbenzofurans were characterized by ^1^H-NMR, ^13^C-NMR and HRMS. The obtained results showed consistency with the expected structures and formulas of the targeted products. The ^1^H-NMR spectra of 2-salicyloylbenzofurans displayed a single signal at the range of δ 7.70–7.30 ppm, which confirmed the formation of benzofuran heterocycle.

### 2.2. Evaluation of Antibacterial Activity

The newly synthesized compounds were preliminarily screened for in vitro qualitative (zone of inhibition) and quantitative (MIC) antibacterial activities against three Gram-(+) strains (MSSA ATCC 29213, MRSA ATCC 43300, and *S. faecalis* ATCC 29212) and one Gram-(–) strain (*E. coli* ATCC 25922) by slight modified agar well diffusion assay [23,24] and agar dilution method [25]. Dimethyl sulfoxide (DMSO) and potent antibacterial drugs ampicillin, cefuroxime and vancomycin were used as solvent control and standards, respectively. The diameters of the inhibition zone (mm) corresponding to the MICs (mM) are presented in Table 1.

The 2-methoxybenzoylbenzofuran **4** showed week antibacterial activity against *E. coli* and *S. faecalis* with inhibition zone diameters of 9.5 mm. Similarly, the respective phenolic compound, 2-salicyloylbenzofuran **5**, showed week antibacterial activity against three Gram-(+) bacterial strains with inhibition zone diameters in the range of 9–13 mm (MICs > 2.59 mM) while completely lost activity against *E. coli*. These preliminary results showed that compound **4** and **5** were considered poor antibacterial agents. From the literature, we found that the substituent at the aroyl-C2 moiety was identified as the important characteristic feature for antibacterial activity [7,8,9,10,11]. Therefore, in order to improve the antibacterial activity of 2-salicyloylbenzofurans, we focused on the replacing of 2-OCH_3_ and 2-OH group by different ether residues. Amongst the obtained ethers, compounds **7a**–**f** except **7c** showed some slight improvements of antibacterial activity with inhibition zone diameters in the range of 8–18 mm (MICs > 1.84 mM) against Gram-(+) and/or Gram-(–) strains. Meanwhile, the 2-salicyloylbenzofuran derivatives **8f**–**h** bearing carboxylic acid group on the ether residue significantly increased their antibacterial activities against three Gram-(+) bacterial strains (*S. faecalis*, MSSA and MRSA) as compared to the parent derivatives **4** and **5** with the MIC values = 0.06–0.55 mM. Compound **8h** showed the most potent antibacterial activity with MIC values = 0.06–0.12 mM against three Gram-(+) bacterial strains. In addition, these 2-salicyloylbenzofurans also displayed inhibitory activity against MRSA with the best MICs = 0.14, 0.27, and 0.12 mM for compound **8f**–**h**, respectively. However, compounds **8f**–**h** were completely inactive against Gram-(–) *E. coli* at the tested concentration. It was found from these results that the carboxylic acid group seems to be an essential part of the pharmacophore required for inhibitory activity against Gram-(+) bacterial strains since the others including nitrile **7c**, amide **7e**, and ester **7f** generally possessed very poor or no antibacterial activity. It was also suggested that the –COOH group keeps an important role for a potential hydrogen bonding as hydrogen bond donor functions with hydrophilic properties.

**Figure 2:**
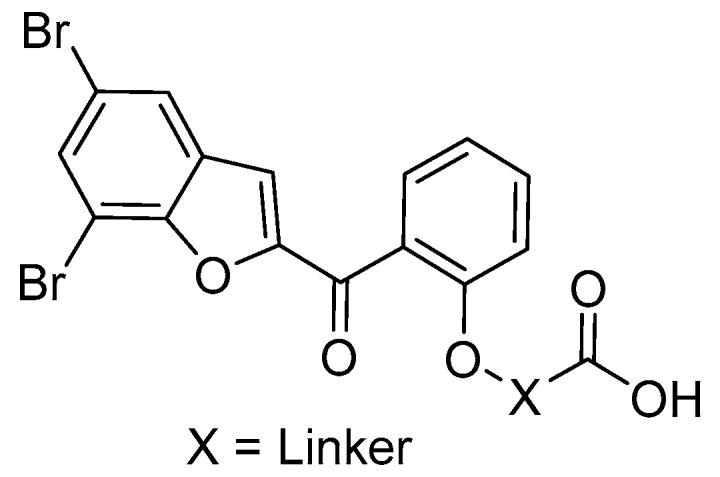
The most potent scaffold for anti-Gram-(+) bacterial strains activity.

We next investigated the potential impact of substituents on the benzofuran heterocycle by replacing bromine atoms with different substituents (**14a**–**f**). The antibacterial activity showed compounds **14a**–**f** decreased their bioactivity with MIC values ≥ 0.70 mM as compared to compound **8f**. These results indicated that bromine atoms may serve as potential halogen bond donor functions with lipophilic properties that are consistent with other research [7]. Besides, most of these derivatives were also inactive against *E. coli* (except compound **14d**, MIC = 3.10 mM) that again confirmed that the presence of carboxylic acid group on the salicyloyl moiety seems to be favor for inhibitory activity against Gram-(+) strains. Overall, the results of this study suggested that the 5,7-dibromo-2-salicyloylbenzofuran bearing carboxylic group via ether linker moiety (Figure 2) may become the promising scaffold for the follow-up research to discover novel antibacterial agents, particularly against Gram-(+) bacterial strains including MRSA, a main cause of a variety of community and healthcare-associated infections.

## 3. Experimental Section

### 3.1. Chemicals and Instruments

All starting materials were purchased from commercial sources such as Acros Organics-Thermo Fisher Scientific (Geel, Belgium), TCI Chemicals (Tokyo, Japan) and used without further purification. Thin-layer chromatography (TLC) was carried out on aluminum-supported silica gel plates (Merck 60F 254, Darmstadt, Germany) with visualization of components by UV light (254 nm). Column chromatography was carried out on silica gel (0.04–0.063 mm, 230–400 mesh ASTM, Merck, Darmstadt, Germany). The melting points were recorded on a Gallenkamp apparatus (Sanyo Gallenkamp, Southborough, UK) and were uncorrected. NMR spectra were recorded in CDCl_3_ or DMSO-*d*_6_ using a Bruker Avance 500 MHz spectrometer (Bruker Corporation, Billerica, MA, USA). Chemical shifts (δ) are reported in ppm related to internal tetramethylsilane (TMS) and coupling constant (*J*) are reported in Hertz (Hz). Mass spectra were recorded on a Shimadzu LCMS-IT-TOF Mass spectrometer (Shimadzu Scientific Instruments, Kyoto, Japan).

Three Gram-(+) strains (MSSA ATCC 29213, MRSA ATCC 43300, *S. faecalis* ATCC 29212) and one Gram-(–) strain (*E. coli* ATCC 25922) were preserved and activated at our Department of Microbiology and Parasitology. The bacterial culture media were purchased from Merck (Darmstadt, Germany) in which Tryptic Soy Agar (TSA) was used to isolate and preserve bacterial strains, Tryptic Soy Broth (TSB) was used as bacteria-activating medium and Muller-Hinton Agar (MHA) was used for antimicrobial susceptibility testing. Dimethyl sulfoxide (DMSO) from Merck and potent antibacterial drugs ampicillin, cefuroxime, and vancomycin (Sigma-Aldrich Pte. Ltd., St. Louis, MO, USA) were used as solvent control and standards, respectively.

### 3.2. Synthesis

#### 3.2.1. General Procedure for the Synthesis of Phenacyl Bromides **2**, **11**

The round-bottom flask containing acetophenone derivative **1**, **10** (4 mmol) and PTSA (0.076 g, 0.4 mmol) was heated to 60 °C to turn the reaction mixture into a paste and NBS (0.854 g, 4.8 mmol) then added slowly. After 15 min, the reaction mixture was cooled to room temperature and water (20 mL) added. The crude product was extracted with dichloromethane (2 × 20 mL), dried over anhydrous Na_2_SO_4_ and purified by crystallization from *n*-hexane–dichloromethane to give pure product.

*2-Methoxyphenacyl bromide* (**2**): Yellow solid, soluble in acetone, dichloromethane, chloroform, insoluble in water; yield 83%; m.p.: 43–45 °C; ^1^H-NMR (500 MHz, CDCl_3_) (δ, ppm): 7.78 (dd, 1H, *J* = 1.5, 7.5 Hz, Ar–H), 7.51–7.47 (m, 1H, Ar–H), 7.02–6.96 (m, 2H, Ar–H), 4.57 (s, 2H, CH_2_), 3.93 (s, 3H, OCH_3_). This result showed consistency with the data in literature [26].

*Ethyl 2-(2-(2-bromoacetyl)phenoxy)acetate* (**11**): White solid, soluble in dichloromethane, chloroform, insoluble in water; yield 74%; m.p.: 75–76 °C; ^1^H-NMR (500 MHz, CDCl_3_) *(*δ, ppm): 7.85 (dd, 1H, *J* = 1.5, 7.5 Hz, Ar–H), 7.52–7.49 (m, 1H, Ar–H), 7.11–7.08 (m, 1H, Ar–H), 6.86 (d, 1H, *J* = 8.0 Hz, Ar–H), 4.79 (s, 2H, CH_2_), 4.74 (s, 2H, CH_2_), 4.30 (q, 2H, *J* = 7.0 Hz, CH_2_CH_3_), 1.33 (t, 3H, *J* = 7.0 Hz, CH_2_CH_3_); ^13^C-NMR (125 MHz, CDCl_3_) (δ, ppm): 192.4 (C=O_ketone_), 167.7 (C=O_ester_), 156.6, 134.5, 131.8, 125.5, 122.1, 112.2, 65.7 (CH_2_COOCH_2_CH_3_), 61.7 (CH_2_COOCH_2_CH_3_), 37.8 (CH_2_Br), 14.1 (CH_3_); HRMS (ESI) *m*/*z* 301.0028 [M + H]^+^, calculated for (C_12_H_14_BrO_4_): 301.0075.

#### 3.2.2. General Procedure for the Synthesis of 2-Salicyloylbenzofurans **4**, **13a**–**f**

In a round-bottom flask containing DMAP (0.012 g, 0.1 mmol) and Na_2_CO_3_ (0.159 g, 1.5 mmol) in water (10 mL), phenacyl bromide **2** or **11** (1 mmol) and salicylaldehyde derivative **3**, **12a**–**f** (1 mmol) were added. The resulting mixture was stirred at 80 °C for 5 h. The mixture was extracted with CH_2_Cl_2_ (3 × 15 mL), and then washed with water (30 mL). The organic layers were combined, dried over anhydrous Na_2_SO_4_ and evaporated under vacuum. The obtained residue was purified by column chromatography on silica gel (*n*-hexane:dichloromethane = 1:1, *v*/*v*) to give pure product.

*5,7-Dibromo-2-(2-methoxybenzoyl)benzofuran* (**4**): Yellow solid, soluble in dichloromethane, chloroform, insoluble in water; yield 72%; m.p. 175–176 °C; ^1^H-NMR (500 MHz, CDCl_3_) (δ, ppm): 7.76–7.75 (m, 2H, Ar–H), 7.55–7.51 (m, 2H, Ar–H), 7.34 (s, 1H, Ar–H), 7.08 (t, 1H, *J* = 7.5 Hz, Ar–H), 7.04 (d, 1H, *J* = 8.5 Hz, Ar–H), 3.81 (s, 3H, OCH_3_); ^13^C-NMR (125 MHz, CDCl_3_) (δ, ppm): 184.3 (C=O), 158.1, 154.7, 152.0, 133.2, 133.1, 130.0, 129.7, 127.4, 124.8, 120.6, 116.8, 114.4, 111.9, 105.9, 55.9 (OCH_3_); HRMS (ESI) *m*/*z* 410.9169 [M + H]^+^, calculated for (C_16_H_11_Br_2_O_3_): 410.9054.

*Ethyl 2-(2-(benzofuran-2-carbonyl)phenoxy)acetate* (**13a**): Yellow brown oil, soluble in dichloromethane, chloroform, insoluble in water; yield 60%; ^1^H-NMR (500 MHz, CDCl_3_) (δ, ppm): 7.68 (d, 1H, *J* = 8.0 Hz, Ar–H), 7.59 (dd, 1H, *J* = 1.0, 8.5 Hz, Ar–H), 7.54 (dd, 1H, *J* = 2.0, 7.5 Hz, Ar–H), 7.50–7.45 (m, 3H, Ar–H), 7.29 (td, 1H, *J* = 0.5, 7.5 Hz, Ar–H), 7.13 (td, 1H, *J* = 1.0, 7.5 Hz, Ar–H), 6.90 (d, 1H, *J* = 8.0 Hz, Ar–H), 4.63 (s, 2H, CH_2_), 4.15 (q, 2H, *J* = 7.0 Hz, CH_2_CH_3_), 1.19 (t, 3H, *J* = 7.0 Hz, CH_2_CH_3_); ^13^C-NMR (125 MHz, CDCl_3_) (δ, ppm): 184.5 (C=O_ketone_), 168.3 (C=O_ester_), 156.0, 155.8, 152.9, 132.4, 130.0, 128.6, 128.3, 127.2, 123.7, 123.4, 121.7, 116.8, 112.8, 112.5, 66.0 (CH_2_COOCH_2_CH_3_), 61.3 (CH_2_COOCH_2_CH_3_), 14.0 (CH_3_); HRMS (ESI) *m*/*z* 325.1027 [M + H]^+^, calculated for (C_19_H_17_O_5_): 325.1076.

*Ethyl 2-(2-(5-methylbenzofuran-2-carbonyl)phenoxy)acetate* (**13b**): Yellow brown oil, soluble in dichloromethane, chloroform, insoluble in water; yield 57%; ^1^H-NMR (500 MHz, CDCl_3_) (δ, ppm): 7.53 (dd, 1H, *J* = 1.5, 7.5 Hz, Ar–H), 7.49–7.44 (m, 3H, Ar–H), 7.39 (s, 1H, Ar–H), 7.28 (dd, 1H, *J* = 1.5, 8.5 Hz, Ar–H), 7.12 (t, 1H, *J* = 7.5 Hz, Ar–H), 6.90 (d, 1H, *J* = 8.5 Hz, Ar–H), 4.62 (s, 2H, CH_2_), 4.15 (q, 2H, *J* = 7.0 Hz, CH_2_CH_3_), 2.44 (s, 3H, CH_3_), 1.20 (t, 3H, *J* = 7.0 Hz, CH_2_CH_3_); ^13^C-NMR (125 MHz, CDCl_3_) (δ, ppm): 182.4 (C=O_ketone_), 171.1 (C=O_ester_), 154.7, 153.8, 152.0, 135.5, 133.5, 132.9, 129.7, 129.6, 125.0, 117.0, 115.6, 114.7, 113.9, 105.9, 73.5 (CH_2_COOCH_2_CH_3_), 61.6 (CH_2_COOCH_2_CH_3_), 18.2 (CH_3_), 14.1 (CH_2_CH_3_); HRMS (ESI) *m*/*z* 339.1222 [M + H]^+^, calculated for (C_20_H_19_O_5_): 339.1232.

*Ethyl 2-(2-(5-methoxybenzofuran-2-carbonyl)phenoxy)acetate* (**13c**): Yellow brown oil, soluble in dichloromethane, chloroform, insoluble in water; yield 66%; ^1^H-NMR (500 MHz, CDCl_3_) (δ, ppm): 7.53 (dd, 1H, *J* = 1.5, 7.5 Hz, Ar–H), 7.50–7.46 (m, 2H, Ar–H), 7.41 (s, 1H, Ar–H), 7.13 (d, 1H, *J* = 7.0 Hz, Ar–H), 7.11–7.06 (m, 2H, Ar–H), 6.90 (d, 1H, *J* = 8.5 Hz, Ar–H), 4.63 (s, 2H, CH_2_), 4.16 (q, 2H, *J* = 7.0 Hz, CH_2_CH_3_), 3.84 (s, 3H, OCH_3_), 1.20 (t, 3H, *J* = 7.0 Hz, CH_2_CH_3_); ^13^C-NMR (125 MHz, CDCl_3_) (δ, ppm): 183.7 (C=O_ketone_), 168.4 (C=O_ester_), 161.2, 157.7, 155.7, 152.5, 132.1, 129.9, 128.9, 123.7, 121.6, 120.6, 117.7, 114.3, 112.8, 95.6, 66.0 (CH_2_COOCH_2_CH_3_), 61.3 (CH_2_COOCH_2_CH_3_), 55.7 (OCH_3_), 14.0 (CH_2_CH_3_); HRMS (ESI) *m*/*z* 355.1165 [M + H]^+^, calculated for (C_20_H_19_O_6_): 355.1182.

*Ethyl 2-(2-(5-chlorobenzofuran-2-carbonyl)phenoxy)acetate* (**13d**): Yellow brown oil, soluble in dichloromethane, chloroform, insoluble in water; yield 70%; ^1^H-NMR (500 MHz, CDCl_3_) (δ, ppm): 7.65 (d, 1H, *J* = 7.0 Hz, Ar–H), 7.54–7.48 (m, 3H, Ar–H), 7.43 (d, 1H, *J* = 0.5 Hz, Ar–H), 7.41 (dd, 1H, *J* = 2.0, 8.5 Hz, Ar–H), 7.14–7.11 (m, 1H, Ar–H), 6.89 (d, 1H, *J* = 8.5 Hz, Ar–H), 4.62 (s, 2H, CH_2_), 4.16 (q, 2H, *J* = 7.0 Hz, CH_2_CH_3_), 1.21 (t, 3H, *J* = 7.0 Hz, CH_2_CH_3_); ^13^C-NMR (125 MHz, CDCl_3_) (δ, ppm): 184.4 (C=O_ketone_), 168.2 (C=O_ester_), 155.8, 154.2, 154.0, 132.8, 132.7, 130.1, 129.3, 128.5, 128.3, 122.7, 121.8, 115.6, 113.6, 112.7, 65.8 (CH_2_COOCH_2_CH_3_), 61.4 (CH_2_COOCH_2_CH_3_), 14.0 (CH_3_); HRMS (ESI) *m*/*z* 359.0672 [M + H]^+^, calculated for (C_19_H_16_BrO_5_): 359.0686.

*Ethyl 2-(2-(5-bromobenzofuran-2-carbonyl)phenoxy)acetate* (**13e**): Yellow brown oil, soluble in dichloromethane, chloroform, insoluble in water; yield 72%; ^1^H-NMR (500 MHz, CDCl_3_) (δ, ppm): 7.82 (d, 1H, *J* = 1.5 Hz, Ar–H), 7.56–7.46 (m, 4H, Ar–H), 7.43 (s, 1H, Ar–H), 7.13 (t, 1H, *J* = 7.5 Hz, Ar–H), 6.89 (d, 1H, *J* = 8.0 Hz, Ar–H), 4.62 (s, 2H, CH_2_), 4.16 (q, 2H, *J* = 7.0 Hz, CH_2_CH_3_), 1.21 (t, 3H, *J* = 7.0 Hz, CH_2_CH_3_); ^13^C-NMR (125 MHz, CDCl_3_) (δ, ppm): 183.9 (C=O_ketone_), 168.1 (C=O_ester_), 156.1, 154.4, 151.9, 133.1, 133.0, 130.3, 129.7, 128.0, 124.9, 121.9, 116.7, 115.0, 112.8, 105.8, 66.0 (CH_2_COOCH_2_CH_3_), 61.4 (CH_2_COOCH_2_CH_3_), 14.0 (CH_3_); HRMS (ESI) *m*/*z* 403.0168 [M + H]^+^, calculated for (C_19_H_16_BrO_5_): 403.0181.

*Ethyl 2-(2-(5,7-dichlorobenzofuran-2-carbonyl)phenoxy)acetate* (**13f**): Yellow brown oil, soluble in dichloromethane, chloroform, insoluble in water; yield 68%; ^1^H-NMR (500 MHz, CDCl_3_) (δ, ppm): 7.57 (d, 1H, *J* = 2.0 Hz, Ar–H), 7.56 (dd, 1H, *J* = 2.0, 7.5 Hz, Ar–H), 7.53–7.50 (m, 1H, Ar–H), 7.49 (s, 1H, Ar–H), 7.46 (d, 1H, *J* = 2.0 Hz, Ar–H), 7.14 (td, 1H, *J* = 1.0, 7.5 Hz, Ar–H), 6.91 (d, 1H, *J* = 8.5 Hz, Ar–H), 4.64 (s, 2H, CH_2_), 4.13 (q, 2H, *J* = 7.0 Hz, CH_2_CH_3_), 1.19 (t, 3H, *J* = 7.0 Hz, CH_2_CH_3_); ^13^C-NMR (125 MHz, CDCl_3_) (δ, ppm): 184.01 (C=O_ketone_), 168.2 (C=O_ester_), 156.2, 154.7, 150.3, 133.1, 130.4, 129.6, 129.5, 128.1, 127.9, 122.0, 121.3, 118.5, 115.1, 112.9, 66.0 (CH_2_COOCH_2_CH_3_), 61.4 (CH_2_COOCH_2_CH_3_), 14.0 (CH_3_); HRMS (ESI) *m*/*z* 393.0246 [M + H]^+^, calculated for (C_19_H_15_Cl_2_O_5_): 393.0297.

#### 3.2.3. Synthesis of 5,7-Dibromo-2-salicyloylbenzofuran **5**

Anhydrous AlCl_3_ (0.267 g, 2.0 mmol) was added to a solution of substrate **4** (0.410 g, 1.0 mmol) in CH_2_Cl_2_ (10 mL) in one portion at 0 °C. The reaction mixture was stirred at the same temperature for 5 min. then warmed up to room temperature and stirred for a further 3 h. The reaction mixture was poured into cold water (20 mL) and extracted with CH_2_Cl_2_ (2 × 20 mL). The combined organic layer was washed successively with saturated NaHCO_3_ and brine, and then dried over anhydrous Na_2_SO_4_. The solvent was evaporated under vacuum and the crude product was purified by column chromatography (*n*-hexan:dichloromethane = 1:1, *v*/*v*) to obtain product as yellow solid. Yield 89%, soluble in dichloromethane, chloroform, insoluble in water; m.p. 132–134 °C; ^1^H-NMR (500 MHz, CDCl_3_) (δ, ppm): 12.04 (s, 1H, OH), 8.47 (dd, 1H, *J* = 1.5, 6.5 Hz, Ar–H), 7.82 (d, 1H, *J* = 1.5 Hz, Ar–H), 7.79 (d, 1H, *J* = 2.0 Hz, Ar–H), 7.67 (s, 1H, Ar–H), 7.59–7.55 (m, 1H, Ar–H), 7.08 (dd, 1H, *J* = 1.0, 7.5 Hz, Ar–H), 7.05–7.02 (m, 1H, Ar–H); ^13^C-NMR (125 MHz, CDCl_3_) (δ, ppm): 185.9 (C=O), 164.1, 154.0, 152.3, 137.3, 133.5, 131.9, 129.2, 124.9, 119.6, 118.8, 118.9, 117.5, 115.6, 106.0; HRMS (ESI) *m*/*z* 394.8756 [M − H]^−^, calculated for (C_15_H_7_Br_2_O_3_): 394.8741.

#### 3.2.4. General Procedure for the Synthesis of *O*-ether Derivatives **7a**–**h**, **10**

A mixture of **5** or **9** (1 mmol), halogenated material **6a**–**h** (1.2 mmol) (**6a**: ethyl bromide, **6b**: allyl bromide; **6c**: 2-bromoacetonitrile, **6d**: 2-chlorobenzyl chloride, **6e**: 2-chloro-*N*-(2,6-dimethylphenyl)acetamide, **6f**: ethyl chloroacetate, **6g**: ethyl 2-bromopropanoate, **6h**: methyl 5-(chloromethyl)furan-2-carboxylate) and anhydrous K_2_CO_3_ (0.276 g, 2.0 mmol) in dry acetone (10 mL) was refluxed for 8 h. The reaction mixture was cooled to room temperature and the solvent was removed under reduced pressure. The residue was triturated with ice water to remove K_2_CO_3_ and then extracted with dichloromethane (3 × 15 mL). The organic layer was dried over anhydrous Na_2_SO_4_, evaporated under vacuum to dryness. The obtained residue was purified by column chromatography using *n*-hexane and dichloromethane (1:1) as eluent to give pure product.

*5,7-Dibromo-2-(2-ethoxybenzoyl)benzofuran* (**7a**): Yellow solid, soluble in dichloromethane, chloroform, insoluble in water; yield 75%; m.p. 78–80 °C; ^1^H-NMR (500 MHz, CDCl_3_) (δ, ppm): 7.77 (d, 1H, *J* = 1.5 Hz, Ar–H), 7.75 (s, 1H, Ar–H), 7.53–7.50 (m, 2H, Ar–H), 7.37 (s, 1H, Ar–H), 7.08–7.05 (m, 1H, Ar–H), 7.01 (d, 1H, *J* = 8.5 Hz, Ar–H), 4.07 (q, 1H, *J* = 7.0 Hz, CH_2_), 1.12 (t, 3H, *J* = 7.0 Hz, CH_3_); ^13^C-NMR (125 MHz, CDCl_3_) (δ, ppm): 184.7 (C=O), 157.6, 154.9, 151.8, 133.3, 133.0, 130.1, 129.7, 127.5, 124.8, 120.6, 116.8, 113.6, 112.7, 105.8, 64.3 (CH_2_), 14.5 (CH_3_); HRMS (ESI) *m*/*z* 422.9249 [M + H]^+^, calculated for (C_17_H_13_Br_2_O_3_): 422.9231.

*2-(2-Allyloxybenzoyl)-5,7-dibromobenzofuran* (**7b**): Yellow solid, soluble in dichloromethane, chloroform, insoluble in water; yield 79%; m.p. 78–80 °C; ^1^H-NMR (500 MHz, CDCl_3_) (δ, ppm): 7.76 (d, 1H, *J* = 1.5 Hz, Ar–H), 7.74 (d, 1H, *J* = 2.0 Hz, Ar–H), 7.54–7.50 (m, 2H, Ar–H), 7.39 (s, 1H, Ar–H), 7.10–7.07 (m, 1H, Ar–H), 7.03 (d, 1H, *J* = 8.5 Hz, Ar–H), 5.80–5.75 (m, 1H, CH_2_CH=CH_2_), 5.15 (dd, 1H, *J* = 1.5, 16.0 Hz, CH_2_CH=CH_2_), 5.05 (dd, 1H, *J* = 1.5, 10.0 Hz, CH_2_CH=CH_2_), 4.56 (dd, 2H, *J* = 1.5, 3.5 Hz, CH_2_CH=CH_2_); ^13^C-NMR (125 MHz, CDCl_3_) (δ, ppm): 184.5 (C=O), 157.2, 154.8, 151.9, 133.3, 133.1, 132.4, 130.1, 129.7, 127.7, 124.8, 120.9, 117.4, 116.8, 113.8, 113.1, 105.9, 69.5 (CH_2_CH=CH_2_); HRMS (ESI) *m*/*z* 476.8714 [M + K]^+^, calculated for (C_18_H_12_Br_2_KO_3_): 476.8749.

*2-(2-(5,7-Dibromobenzofuran-2-carbonyl)phenoxy)acetonitrile* (**7c**): White solid, soluble in dichloromethane, acetone, insoluble in water; yield 87%; m.p.: 178–179 °C; ^1^H-NMR (500 MHz, CDCl_3_) (δ, ppm): 7.78 (d, 1H, *J* = 1.5 Hz, Ar–H), 7.77 (d, 1H, *J* = 1.5 Hz, Ar–H), 7.62–7.59 (m, 2H, Ar–H), 7.34 (s, 1H, Ar–H), 7.26 (t, 1H, *J* = 8.0 Hz, Ar–H), 7.19 (d, 1H, *J* = 8.5 Hz, Ar–H), 4.81 (s, 2H, CH_2_); ^13^C-NMR (125 MHz, CDCl_3_) (δ, ppm): 183.1 (C=O), 154.9, 154.0, 152.1, 133.6, 133.4, 130.5, 129.5, 128.6, 125.0, 123.5, 117.2, 115.2, 114.6, 114.1, 105.9, 54.8 (CH_2_); HRMS (ESI) *m*/*z* 433.8770 [M − H]^−^, calculated for (C_17_H_8_Br_2_NO_3_): 433.8851.

*2-(2-(2-Chlorobenzyloxy)benzoyl)-5,7-dibromobenzofuran* (**7d**): Yellow solid, soluble in dichloromethane, chloroform, insoluble in water; yield 77%; m.p.: 125–127 °C; ^1^H-NMR (500 MHz, CDCl_3_) (δ, ppm): 7.71 (s, 1H, Ar–H), 7.67 (s, 1H, Ar–H), 7.59–7.53 (m, 2H, Ar–H), 7.39 (s, 1H, Ar–H), 7.27 (d, 1H, *J* = 8.5 Hz, Ar–H), 7.14–7.11 (m, 4H, Ar–H), 6.99 (m, 1H, Ar–H), 5.20 (s, 2H, CH_2_); ^13^C-NMR (125 MHz, CDCl_3_) (δ, ppm): 184.5 (C=O), 157.1, 154.7, 151.9, 133.9, 133.47, 133.0, 131.9, 130.3, 129.6, 129.1, 128.8, 127.8, 127.6, 126.8, 124.7, 121.3, 116.8, 113.9, 113.3, 105.9, 67.9 (CH_2_ ); HRMS (ESI) *m*/*z* 542.8563 [M + Na]^+^, calculated for (C_22_H_13_Br_2_ClNaO_3_): 542.8797.

*2-(2-(5,7-Dibromobenzofuran-2-carbonyl)phenoxy)-N-(2,6-dimethylphenyl)acetamide* (**7e**): Yellow solid, soluble in dichloromethane, chloroform, insoluble in water; yield 75%; m.p.: 177–179 °C; ^1^H-NMR (500 MHz, CDCl_3_) (δ, ppm): 8.65 (s, 1H, NH), 7.76–7.72 (m, 3H, Ar–H), 7.65–7.62 (m, 1H, Ar–H), 7.44 (s, 1H, Ar–H), 7.20 (t, 1H, *J* = 7.5 Hz, Ar–H), 7.16 (d, 1H, *J* = 8.5 Hz, Ar–H), 7.12–7.05 (m, 3H, Ar–H), 4.81 (s, 2H, CH_2_), 2.12 (s, 6H, CH_3_); ^13^C-NMR (125 MHz, CDCl_3_) (δ, ppm): 183.2 (C=O_ketone_), 166.2 (C=O_amide_), 156.3, 153.8, 152.3, 135.3, 134.2, 133.8, 133.0, 133.0, 131.0, 129.3, 129.3, 128.2, 127.4, 126.4, 124.9, 121.7, 117.3, 115.8, 113.4, 106.1, 67.8 (CH_2_), 18.3 (CH_3_); HRMS (ESI) *m*/*z* 579.9592 [M + Na]^+^, calculated for (C_25_H_19_Br_2_NNaO_4_): 579.9558.

*Ethyl 2-(2-(5,7-dibromobenzofuran-2-carbonyl)phenoxy)acetate* (**7f**): White solid, soluble in dichloromethane, acetone, insoluble in water; yield 64%; m.p.: 77–78 °C; ^1^H-NMR (500 MHz, CDCl_3_) (δ, ppm): 7.77 (d, 1H, *J* = 1.5 Hz, Ar–H), 7.74 (d, 1H, *J* = 1.5 Hz, Ar–H), 7.57–7.50 (m, 3H, Ar–H), 7.16–7.13 (m, 1H, Ar–H), 6.91 (d, 1H, *J* = 8.0 Hz, Ar–H), 4.64 (s, 2H, CH_2_COOCH_2_CH_3_), 4.12 (q, 2H, *J* = 7.0 Hz, CH_2_COOCH_2_CH_3_), 1.19 (t, 3H, *J* = 7.0 Hz, CH_3_); ^13^C-NMR (125 MHz, CDCl_3_) (δ, ppm): 184.0 (C=O_ketone_), 168.2 (C=O_ester_), 156.2, 154.4, 152.0, 133.1, 133.1, 130.4, 129.8, 128.1, 124.9, 122.0, 116.8, 115.0, 112.8, 105.9, 66.1 (CH_2_COOCH_2_CH_3_), 61.4 (CH_2_COOCH_2_CH_3_), 14.0 (CH_3_); HRMS (ESI) *m*/*z* 504.9030 [M + Na]^+^, calculated for (C_19_H_14_Br_2_NaO_5_): 504.9085.

*Ethyl 2-(2-(5,7-dibromobenzofuran-2-carbonyl)phenoxy)propanoate* (**7g**): Brown oil, soluble in dichloromethane, chloroform, insoluble in water; yield 60%; ^1^H-NMR (500 MHz, CDCl_3_) (δ, ppm): 7.77–7.75 (m, 2H, Ar–H), 7.57 (s, 1H, Ar–H), 7.53 (d, 1H, *J* = 7.5 Hz, Ar–H), 7.47 (t, 1H, *J* = 8.0 Hz, Ar–H), 7.10 (t, 1H, *J* = 7.5 Hz, Ar–H), 6.83 (d, 1H, *J* = 8.5 Hz, Ar–H), 4.76 (q, 1H, *J* = 6.5 Hz, CH), 4.18 (q, 2H, *J* = 7.0 Hz, CH_2_), 1.37 (d, 3H, *J* = 6.5 Hz, CH_3_CH), 1.24 (t, 3H, *J* = 7.0 Hz, CH_2_CH_3_); ^13^C-NMR (125 MHz, CDCl_3_) (δ, ppm): 184.1 (C=O_ketone_), 171.6 (C=O_ester_), 155.7, 154.4, 151.9, 133.2, 133.0, 130.4, 129.8, 128.1, 124.9, 121.6, 116.8, 115.3, 112.8, 105.9, 73.2 (CH_3_CH), 61.4 (CH_2_CH_3_) , 18.3 (CH_3_CH), 14.1 (CH_2_CH_3_); HRMS (ESI) *m*/*z* 518.9387 [M + Na]^+^, calculated for (C_20_H_16_Br_2_NaO_5_): 518.9242.

*Methyl 5-((2-(5,7-dibromobenzofuran-2-carbonyl)phenoxy)methyl)furan-2-carboxylate* (**7h**): White solid, soluble in dichloromethane, acetone, insoluble in water; yield 77%; m.p.: 122–124 °C; ^1^H-NMR (500 MHz, CDCl_3_) (δ, ppm): 7.75 (d, 1H, *J* = 1.5 Hz, Ar–H), 7.72 (d, 1H, *J* = 1.5 Hz, Ar–H), 7.56–7.52 (m, 2H, Ar–H), 7.36 (s, 1H, Ar–H), 7.14 (t, 1H, *J* = 7.5 Hz, Ar–H), 7.10 (d, 1H, *J* = 8.5 Hz, Ar–H), 6.97 (d, 1H, *J* = 3.5 Hz, Ar–H), 6.24 (d, 1H, *J* = 3.5 Hz, Ar–H), 5.10 (s, 2H, CH_2_), 3.87 (s, 3H, CH_3_); ^13^C-NMR (125 MHz, CDCl_3_) (δ, ppm): 182.5 (C=O_ketone_), 158.7 (C=O_ester_), 155.4, 154.0, 153.0, 151.9, 144.6, 135.8, 133.4, 132.8, 129.7, 129.5, 125.0, 118.5, 117.0, 115.1, 114.6, 114.2, 111.4, 105.9, 63.6 (CH_2_), 52.1 (CH_3_); HRMS (ESI) *m*/*z* 556.9210 [M + Na]^+^, calculated for (C_22_H_14_Br_2_NaO_6_): 556.9034.

*Ethyl 2-(2-acetylphenoxy)acetate* (**10**): White solid, soluble in dichloromethane, chloroform, insoluble in water; yield 77%; m.p.: 46–48 °C; ^1^H-NMR (500 MHz, CDCl_3_) (δ, ppm): 7.76 (dd, 1H, *J* = 1.5, 7.5 Hz, Ar–H), 7.46–7.42 (m, 1H, Ar–H), 7.05 (t, 1H, *J* = 7.5 Hz, Ar–H), 6.83 (d, 1H, *J* = 8.5 Hz, Ar–H), 6.83 (d, 1H, *J* = 8.5 Hz, Ar–H), 4.72 (s, 2H, CH_2_) 4.28 (q, 2H, *J* = 7.0 Hz, CH_2_CH_3_), 2.72 (s, 3H, CH_3_C=O), 1.30 (t, 3H, *J* = 7.0 Hz, CH_2_CH_3_); ^13^C-NMR (125 MHz, CDCl_3_) (δ, ppm): 199.6 (C=O_ketone_), 168.1 (C=O_ester_), 157.0, 133.5, 130.6, 128.9, 121.6, 112.2, 65.5 (CH_2_COOCH_2_CH_3_), 61.5 (CH_2_COOCH_2_CH_3_), 32.0 (CH_3_CO), 14.1 (CH_2_CH_3_); HRMS (ESI) *m*/*z* 223.0963 [M + H]^+^, calculated for (C_12_H_15_O_4_): 223.0970.

#### 3.2.5. General Procedure for the Synthesis of Carboxylic Acid Derivatives of 2-salicyloylbenzofurans **8f**–**h** and **14a**–**f**

To a solution of the ester **7f**–**h**, **13a**–**f** (1 mmol) in a solvent mixture of dichloromethane and methanol (9:1, *v*/*v*, 15 mL) was added a methanolic solution of NaOH 2N (1 mL). After 5–10 min of stirring, the sodium salt of the carboxylic acid started to precipitate (except compound **13a**–**c**, the solid not formed). The mixture was stirred and monitored by TLC (*n*-hexane–dichloromethane = 1:1) until all the ester was consumed (after 30–60 min). 

Purification for product **8f**–**h**, **14d**–**f**: The reaction mixture was filtered under reduced pressure, washed with dichloromethane (10 mL) to obtain white solid that was then dissolved in dimethylformamide (DMF) (15–20 mL). The mixture was cooled, acidified to pH 3–4 with dilute HCl to afford the respective carboxylic acid. The mixture was then filtered, washed with cold water and dried in vacuum-heating oven to obtain the targeted product.

Purification for product **14a**–**c**: The reaction mixture was added water (40 mL) and then extracted with dichloromethane (10 mL × 2) to remove impurities. The water solution was cooled, acidified to pH 3–4 with dilute HCl to afford the respective carboxylic acid that extracted with ethyl acetate (15 mL × 2). The combined organic layer was dried over anhydrous Na_2_SO_4_, and then evaporated under vacuum to dryness. The obtained residue was dried in vacuum-heating oven to obtain the targeted product.

*2-(2-(5,7-Dibromobenzofuran-2-carbonyl)phenoxy)acetic acid* (**8f**): White solid, soluble in dimethyl sulfoxide, ethyl acetate, insoluble in water; yield 70%; m.p. 156–158 °C; ^1^H-NMR (500 MHz, DMSO-*d*_6_) (δ, ppm): 8.03 (s, 1H, Ar–H), 8.01 (s, 1H, Ar–H), 7.65 (s, 1H, Ar–H), 7.60–7.57 (m, 1H, Ar–H), 7.53 (d, 1H, *J* = 7.5 Hz, Ar–H), 7.15–7.14 (m, 2H, Ar–H), 4.72 (s, 2H, CH_2_); ^13^C-NMR (125 MHz, DMSO-*d*_6_) (δ, ppm): 183.65 (C=O_ketone_), 169.5 (C=O_acid_), 155.7, 153.5, 151.4, 133.0, 132.7, 129.8, 129.5, 127.1, 125.7, 121.2, 116.3, 116.3, 113.2, 105.2, 65.1 (CH_2_); HRMS (ESI) *m*/*z* 476.8714 [M + Na]^+^, calculated for (C_17_H_10_Br_2_NaO_5_): 476.8772.

*2-(2-(5,7-Dibromobenzofuran-2-carbonyl)phenoxy)propanoic acid* (**8g**): Yellow solid, soluble in dimethyl sulfoxide, ethyl acetate, insoluble in water; yield 75%; m.p.: 153–155 °C; ^1^H-NMR (500 MHz, DMSO-*d*_6_) (δ, ppm): 8.03 (d, 1H, *J =* 1.5 Hz, Ar–H), 7.99 (d, 1H, *J* = 1.5 Hz, Ar–H), 7.69 (s, 1H, Ar–H), 7.59–7.55 (m, 1H, Ar–H), 7.53–7.51 (m, 1H, Ar–H), 7.13 (t, 1H, *J* = 7.5 Hz, Ar–H), 7.04 (d, 1H, *J* = 8.0 Hz, Ar–H), 4.90 (q, 1H, *J* = 6.5 Hz, CH), 1.24 (d, 3H, *J* = 6.5 Hz, CH_3_); ^13^C-NMR (125 MHz, DMSO-*d*_6_) (δ, ppm): 183.7 (C=O_ketone_), 172.5 (C=O_acid_), 155.4, 153.6, 151.3, 133.1, 132.7, 129.7, 127.2, 125.7, 121.0, 116.3, 116.0, 113.2, 105.2, 72.1 (CH_3_CH), 17.8 (CH_3_); HR-MS (ESI) *m*/*z* 466.8869 [M − H]^−^, calculated for (C_18_H_11_Br_2_O_5_): 466.8953.

*5-((2-(5,7-Dibromobenzofuran-2-carbonyl)phenoxy)methyl)furan-2-carboxylic acid* (**8h**): White solid, soluble in dimethyl sulfoxide, ethyl acetate, insoluble in water; yield 82%; m.p.: 131–133 °C; ^1^H-NMR (500 MHz, DMSO-*d*_6_) (δ, ppm): 8.01 (d, 1H, *J* = 1.5 Hz, Ar–H), 7.97 (d, 1H, *J* = 1.5 Hz, Ar–H), 7.54–7.60 (m, 1H, Ar–H), 7.55 (s, 1H, Ar–H), 7.53 (dd, 1H, *J* = 1.5, 7.5 Hz, Ar–H), 7.40 (d, 1H, *J =* 8.5 Hz, Ar–H), 7.16 (t, 1H, *J* = 7.5 Hz, Ar–H), 7.03 (d, 1H, *J* = 3.5 Hz, Ar–H), 6.49 (d, 1H, *J* = 3.5 Hz, Ar–H), 5.21 (s, 2H, CH_2_); ^13^C-NMR (125 MHz, DMSO-*d*_6_) (δ, ppm): 183.6 (C=O_ketone_), 159.0 (C=O_acid_), 155.7, 153.5, 153.3, 151.3, 145.0, 133.2, 132.8, 129.7, 129.6, 127.4, 125.8, 121.4, 118.1, 116.4, 116.1, 113.8, 112.0, 105.2, 62.4 (CH_2_); HRMS (ESI) *m*/*z* 518.8745 [M − H]^−^, calculated for (C_21_H_11_Br_2_O_6_): 518.8902.

*2-(2-(Benzofuran-2-carbonyl)phenoxy)acetic acid* (**14a**): Yellow solid, soluble in dimethyl sulfoxide, ethyl acetate, insoluble in water; yield 72%; m.p.: 115–117 °C; ^1^H-NMR (500 MHz, CDCl_3_) (δ, ppm): 7.75 (d, 1H, *J* = 7.5 Hz, Ar–H), 7.72 (d, 1H, *J* = 8.0 Hz, Ar–H), 7.63–7.58 (m, 2H, Ar–H), 7.54–7.51 (m, 2H, Ar–H), 7.34 (t, 1H, *J* = 7.0 Hz, Ar–H), 7.20 (t, 1H, *J* = 7.5 Hz, Ar–H), 7.08 (d, 1H, *J* = 8.5 Hz, Ar–H), 4.79 (s, 2H, CH_2_); ^13^C-NMR (125 MHz, CDCl_3_) (δ, ppm): 184.5 (C=O_ketone_), 170.3 (C=O_acid_), 156.6, 156.4, 152.0, 134.0, 131.2, 129.2, 126.9, 124.2, 123.6, 122.3, 118.6, 114.8, 112.6, 67.1 (CH_2_); HRMS (ESI) *m*/*z* 297.0749 [M + H]^+^, calculated for (C_17_H_13_O_5_): 297.0763.

*2-(2-(5-Methylbenzofuran-2-carbonyl)phenoxy)acetic acid* (**14b**): Yellow solid, soluble in dimethyl sulfoxide, ethyl acetate, chloroform, insoluble in water; yield 66%; m.p.: 122–124 °C; ^1^H-NMR (500 MHz, CDCl_3_) (δ, ppm): 7.74 (dd, 1H, *J* = 1.5, 7.5 Hz, Ar–H), 7.61–7.57 (m, 1H, Ar–H), 7.51–7.46 (m, 3H, Ar–H), 7.34 (dd, 1H, *J* = 1.5, 7.5 Hz, Ar–H), 7.20 (t, 1H, *J* = 7.5 Hz, Ar–H), 7.08 (d, 1H, *J* = 8.0 Hz, Ar–H), 4.79 (s, 2H, CH_2_), 2.46 (s, 3H, CH_3_); ^13^C-NMR (125 MHz, CDCl_3_) (δ, ppm): 184.5 (C=O_ketone_), 170.3 (C=O_acid_), 156.6, 155.1, 152.1, 134.0, 133.9, 131.3, 130.9, 127.1, 126.9, 123.0, 122.3, 118.5, 114.9, 112.2, 67.2 (CH_2_), 21.3 (CH_3_); HRMS (ESI) *m*/*z* 311.0909 [M + H]^+^, calculated for (C_18_H_15_O_5_): 311.0919.

*2-(2-(5-Methoxybenzofuran-2-carbonyl)phenoxy)acetic acid* (**14c**): Yellow solid, soluble in dimethyl sulfoxide, ethyl acetate, chloroform, insoluble in water; yield 73%; m.p.: 168–171 °C; ; ^1^H-NMR (500 MHz, CDCl_3_) (δ, ppm): 7.68 (dd, 1H, *J* = 1.0, 7.5 Hz, Ar–H), 7.57–7.54 (m, 2H, Ar–H), 7.44 (s, 1H, Ar–H), 7.17 (t, 1H, *J* = 7.5 Hz, Ar–H), 7.07–7.06 (m, 2H, Ar–H), 6.95 (dd, 1H, *J* = 2.0, 8.5 Hz, Ar–H), 4.77 (s, 2H, CH_2_), 3.97 (s, 3H, OCH_3_); ^13^C-NMR (125 MHz, CDCl_3_) (δ, ppm): 183.8 (C=O_ketone_), 170.6 (C=O_acid_), 161.9, 158.3, 156.5, 151.6, 133.7, 131.0, 127.2, 124.0, 122.2, 120.4, 119.7, 115.0, 114.8, 95.5, 67.2 (CH_2_), 55.8 (OCH_3_); HRMS (ESI) *m*/*z* 327.0853 [M + H]^+^, calculated for (C_18_H_15_O_6_): 327.0869.

*2-(2-(5-Chlorobenzofuran-2-carbonyl)phenoxy)acetic acid* (**14d**): White solid, soluble in dimethyl sulfoxide, ethyl acetate, insoluble in water; yield 79%; m.p.: 175–177 °C; ^1^H-NMR (500 MHz, DMSO-*d*_6_) (δ, ppm): 7.86 (s, 1H, Ar–H), 7.76 (d, 1H, *J* = 9.0 Hz, Ar–H), 7.56–7.49 (m, 4H, Ar–H), 7.14–7.11 (m, 2H, Ar–H), 4.71 (s, 2H, CH_2_); ^13^C-NMR (125 MHz, DMSO-*d*_6_) (δ, ppm): 184.0 (C=O_ketone_), 169.6 (C=O_acid_), 155.6, 153.7, 153.5, 132.6, 129.3, 128.5, 128.4, 128.3, 127.5, 123.0, 121.0, 115.9, 114.0, 113.0, 64.9 (CH_2_); HRMS (ESI) *m*/*z* 331.0356 [M + H]^+^, calculated for (C_17_H_12_ClO_5_): 331.0373.

*2-(2-(5-Bromobenzofuran-2-carbonyl)phenoxy)acetic acid* (**14e**): White solid, soluble in dimethyl sulfoxide, ethyl acetate, insoluble in water; yield 81%; m.p.: 145–147 °C; ^1^H-NMR (500 MHz, DMSO-*d*_6_) (δ, ppm): 8.00 (d, 1H, *J* = 2.0 Hz, Ar–H), 7.71 (d, 1H, *J* = 8.5 Hz, Ar–H), 7.70 (dd, 1H, *J* = 2.0, 8.5 Hz, Ar–H), 7.58–7.54 (m, 2H, Ar–H), 7.49 (dd, 1H, *J* = 1.5, 7.5 Hz, Ar–H), 7.14–7.11 (m, 2H, Ar–H), 4.70 (s, 2H, CH_2_); ^13^C-NMR (125 MHz, DMSO-*d*_6_) (δ, ppm): 183.9 (C=O_ketone_), 169.6 (C=O_acid_), 155.6, 154.0, 153.2, 132.6, 131.1, 129.3, 129.1, 127.5, 126.0, 121.0, 116.1, 115.7, 114.4, 113.0, 64.9 (CH_2_); HRMS (ESI) *m*/*z* 374.9850 [M + H]^+^, calculated for (C_17_H_12_BrO_5_): 374.9868.

*2-(2-(5,7-Dichlorobenzofuran-2-carbonyl)phenoxy)acetic acid* (**14f**): White solid, soluble in dimethyl sulfoxide, ethyl acetate, insoluble in water; yield 80%; m.p.: 162–164 °C; ^1^H-NMR (500 MHz, DMSO-*d*_6_) (δ, ppm): 7.85 (d, 1H, *J* = 2.0 Hz, Ar–H), 7.80 (d, 1H, *J* = 1.5 Hz, Ar–H), 7.64 (s, 1H, Ar–H), 7.58 (td, 1H, *J* = 1.5, 8.5 Hz, Ar–H), 7.52 (dd, 1H, *J* = 1.5, 7.5 Hz, Ar–H), 7.16–7.13 (m, 2H, Ar–H), 4.71 (s, 2H, CH_2_); ^13^C-NMR (125 MHz, DMSO-*d*_6_) (δ, ppm): 183.6 (C=O_ketone_), 169.5 (C=O_acid_), 155.7, 153.9, 149.6, 133.0, 129.5, 129.4, 128.5, 127.6, 127.1, 122.2, 121.1, 117.2, 116.2, 113.2, 65.1 (CH_2_); HRMS (ESI) *m*/*z* 364.9960 [M + H]^+^, calculated for (C_17_H_11_Cl_2_O_5_): 364.9984.

### 3.3. Antibacterial Activity

#### 3.3.1. Measurement of the Zones of Inhibition

The in vitro qualitative (zone of inhibition) against three Gram-(+) strains (MSSA ATCC 29213, MRSA ATCC 43300, *S. faecalis* ATCC 29212) and one Gram-(–) strain (*E. coli* ATCC 25922) was performed for 2-salicyloylbenzofuran derivatives by modified agar well diffusion assay [23,24]. DMSO and potent antibacterial drugs ampicillin (10 µg/well), cefuroxime (7.5 µg/well), vancomycin (30 µg/well) were used as solvent control and standards, respectively. The test compounds were dissolved first in DMSO with the concentration of 2048 µg/mL. Fifteen milliliters of the molten MHA (45 °C) were poured into sterile Petri dishes. The cell suspensions containing 1.5 × 10^8^ CFU/mL cells were prepared and spread onto the surface of the agar plates using sterile swab sticks. Once the plates had been aseptically dried, 6 mm wells were bored using a sterile cork borer and samples (60 µL) were placed into the wells. The test plates were incubated at 37 °C for 24 h. Antibacterial activity was evaluated by measuring the diameter (mm) inhibitory zones around wells. The tests were performed in duplicate.

#### 3.3.2. Measurement of MIC Values

The quantitative (MIC) antibacterial activity against three Gram-(+) strains (MSSA ATCC 29213, MRSA ATCC 43300, *S. faecalis* ATCC 29212) and one Gram-(–) strain (*E. coli* ATCC 25922) was determined by agar dilution method as per CLSI recommendation on MHA containing 2-fold serial dilutions of antibiotics and selectively active 2-salicyloylbenzofurans [25]. The compounds in the test medium were prepared at the required quantities of 1024, 512, 256, 128, 64, 32, 16, and 8 µg/mL concentrations and the standard drugs at 32, 16, 8, 4, 2, 1, 0.5, 0.25, 0.125, and 0.0625 µg/mL with MHA. The final inoculum size was 10^4^ CFU/mL for the antibacterial assay. All samples were tested in duplicate. The test plates were incubated at 37 °C for 24 h. The MIC (expressed in µg/mL) was taken as the minimum concentration of the dilution with no growth of microorganisms. The concentration of the solvents used in the following assays was maintained at 2–5% so that no inhibition of microorganisms or interference occurred.

## 4. Conclusions

In summary, we designed, synthesized twenty five 2-salicyloylbenzofuran derivatives and evaluated for their antibacterial activities against three Gram-(+) strains (MSSA ATCC 29213, MRSA ATCC 43300, *S. faecalis* ATCC 29212) and one Gram-(–) strain (*E. coli* ATCC 25922). Most of the 2-salicyloylbenzofurans were found to possess weak to moderate activities against Gram-(+) and/ or Gram-(–) bacteria with the inhibition zone diameters of 8–19 mm and MIC values ≥ 0.06 mM. Amongst the 2-salicyloylbenzofurans bearing carboxylic acid functional group **8f**–**h** and **14a**–**f** displayed the stronger bioactivity and showed the strain-specific to Gram-(+) bacteria with the most potent antibacterial agent **8h** (MICs = 0.06–0.12 mM). In addition, the 2-salicyloylbenzofuran scaffold was found to exhibit activity against MRSA with the best MIC values = 0.14 mM (**8f**) and 0.12 mM (**8h**). The antibacterial results suggested that the substituent bearing carboxylic acid group at salicyloyl-C2 and the bromine atoms on the benzofuran moiety might be the functional necessary for antibacterial activity. Finally, although the antibacterial activities of 2-salicyloylbenzofurans in this study were not very significant, these results initially provide some necessary information for further chemical optimization of this scaffold in the future.

## Figures and Tables

**Table 1:** In-vitro antibacterial activity of 2-salicyloylbenzofuran derivatives.

Compound ^b^	Zone of Inhibition in mm (MIC in mM) ^a^			
	*E. coli*	*S. faecalis*	MSSA	MRSA
**4**	9.5 (>2.50)	9.5 (>2.50)	–	–
**5**	–	9 (>2.59)	10 (>2.59)	13 (>2.59)
**7a**	8 (>2.41)	–	–	12 (>2.41)
**7b**	8 (>2.35)	–	12 (2.35)	16 (2.35)
**7c**	–	–	–	–
**7d**	8(>1.97)	–	–	14 (>1.97)
**7e**	8 (>1.84)	–	18 (>1.84)	–
**7f**	–	–	–	8 (>2.12)
**8f**	–	12 (0.28)	16 (0.14)	14 (0.14)
**8g**	–	11 (0.55)	19 (0.27)	17 (0.27)
**8h**	–	15 (0.12)	17 (0.06)	15 (0.12)
**14a**	–	–	10 (0.86)	11 (>3.46)
**14b**	–	–	9 (1.65)	9 (3.30)
**14c**	–	9 (3.14)	9 (1.57)	9 (1.57)
**14d**	9 (3.10)	9 (>3.10)	9 (1.55)	10 (1.55)
**14e**	–	9 (2.73)	9 (1.36)	11 (1.36)
**14f**	–	11 (1.40)	9 (0.70)	11 (0.70)
**Ampicillin**	35 (0.006)	28 (0.006)	31 (0.0007)	15 (0.05)
**Cefuroxime**	33 (0.02)	–	31 (0.02)	25 (0.04)
**Vancomycin**	23 (0.0014)	30 (0.00004)	30 (0.00004)	31 (0.00004)

^a^ *E. coli*: *Escherichia coli* ATCC 25922; *S. faecalis*: *Streptococcus faecalis* ATCC 29212; MSSA: methicillin-sensitive *Staphylococcus aureus* ATCC 29213; MRSA: methicillin-resistant *Staphylococcus aureus* ATCC 43300; ^b^ other compounds: not tested; (–): not inhibition at tested concentration.
